# Human-specific epigenetic variation in the immunological Leukotriene B4 Receptor (*LTB4R*/BLT1) implicated in common inflammatory diseases

**DOI:** 10.1186/gm536

**Published:** 2014-03-05

**Authors:** Gareth A Wilson, Lee M Butcher, Holly R Foster, Andrew Feber, Christian Roos, Lutz Walter, Grzegorz Woszczek, Stephan Beck, Christopher G Bell

**Affiliations:** 1 Medical Genomics, UCL Cancer Institute, University College London, London, UK; 2 MRC & Asthma UK Centre in Allergic Mechanisms of Asthma, Division of Asthma, Allergy and Lung Biology, King’s College London, London, UK; 3 Genebank of Primates and Primate Genetics Laboratory, German Primate Centre, Leibniz Institute for Primate Research, Göttingen, Germany; 4 Current address: Department of Twin Research & Genetic Epidemiology, St Thomas’ Hospital, King’s College London, London, UK; 5 Current address: Translational Cancer Therapeutics, CR-UK London Research Institute, Lincoln’s Inn Fields, London, UK

## Abstract

**Background:**

Common human diseases are caused by the complex interplay of genetic susceptibility as well as environmental factors. Due to the environment’s influence on the epigenome, and therefore genome function, as well as conversely the genome’s facilitative effect on the epigenome, analysis of this level of regulation may increase our knowledge of disease pathogenesis.

**Methods:**

In order to identify human-specific epigenetic influences, we have performed a novel genome-wide DNA methylation analysis comparing human, chimpanzee and rhesus macaque.

**Results:**

We have identified that the immunological Leukotriene B4 receptor (*LTB4R*, BLT1 receptor) is the most epigenetically divergent human gene in peripheral blood in comparison with other primates. This difference is due to the co-ordinated active state of human-specific hypomethylation in the promoter and human-specific increased gene body methylation. This gene is significant in innate immunity and the LTB4/LTB4R pathway is involved in the pathogenesis of the spectrum of human inflammatory diseases. This finding was confirmed by additional neutrophil-only DNA methylome and lymphoblastoid H3K4me3 chromatin comparative data. Additionally we show through functional analysis that this receptor has increased expression and a higher response to the LTB4 ligand in human versus rhesus macaque peripheral blood mononuclear cells. Genome-wide we also find human species-specific differentially methylated regions (human s-DMRs) are more prevalent in CpG island shores than within the islands themselves, and within the latter are associated with the CTCF motif.

**Conclusions:**

This result further emphasises the exclusive nature of the human immunological system, its divergent adaptation even from very closely related primates, and the power of comparative epigenomics to identify and understand human uniqueness.

## Background

In the past half century there has been a dramatic increase in chronic inflammatory and metabolic common human diseases [1,2]. This is too rapid a time frame to be due to changes in common genetic allele frequency; therefore, the environment of this increasingly urbanized human population is implicated [3]. These environmental effects can influence the genome through the more malleable epigenome [4,5]. Thus, by studying this level of regulation, we may identify the genes and pathways modified by this modern environment that are involved in the pathogenesis of these diseases.

Over a far longer evolutionary term, the environmental pressure experienced since the last common primate ancestor has been a major driver in the human phenotype we see today. Comparative genetic analyses of human [6] with close relatives such as the chimpanzee [7], rhesus macaque [8] and other primates [9-12] have explored these alterations at the sequence level. Accelerated regions have highlighted potentially modified neurological [13,14] and anatomic [15] pathways, human-specific duplications have been implicated in enhanced neuronal migration [16] and human-specific deletions have identified lost regulatory regions [17]. Comparative epigenomics has only recently started to be explored in a number of tissues [18-22]. This regulatory level has a plausible role as a rapid adaptive response mechanism to external change. It is therefore hypothesized to hold insights into recent environmental effects impacting on genomic activity [23,24]. Furthermore, this regulatory change can occur gradually, minimizing effects on viability.

To fully understand human susceptibility to disease we require precise knowledge of what makes us unique. This will be the result of the integration of genetic and epigenetic differences, and their interplay with the environment. The aim of this study was to find the most substantial human species-specific DNA methylome variation in peripheral blood, a tissue type integral to immune responses, by triangulation analysis [25] between human, chimpanzee, and rhesus macaque. The epigenomic mark of DNA methylation is critical for development and is strongly associated with gene regulation. This human-specific DNA methylome variation will be driven by obligatory and facilitative genetic factors, as well as pure epigenetic effects [26]. Genetic divergence in orthologous loci will contribute to methylome variation; by alterations in transcription factor binding sites (TFBSs) that act as methylation determining regions (MDRs) [27]; or associated with CpG density changes, due to gain or loss of species-specific CpGs [28], impacting on CpG island (CpGi) strength [29-31]. The major proportional components of peripheral blood cells are similar between all three species [32-34]; therefore, methylation differences affecting the major cell type fractions or multiple subtypes will be detected. Finally there is the possibility that changes in DNA methylation may be driven as a response to the different environmental stimuli encountered by the primates [35].

The observed methylation state may play an active role in expression, or may passively accumulate due to lack of transcription factor binding, but in either situation is still informative of robust variation in effectors and regulation. Thus, the possible identification of environmentally driven DNA methylation variation between the species could be highly informative in terms of the analysis of the pathophysiology of human disease, particularly if this was able to quantify modern lifestyle disease risk factors [4,36]. The various environmental pressures, bottlenecks, and drift over the separate courses of these species since the last common ancestor of human and chimpanzee (approximately 5 to 6 million years ago (MYA)) [37] will have then left not only a genetic [38] but also an epigenetic human-specific signature. Understanding these modifications will reveal insights into human-specific physiology and potentially human-specific responses to environmental change and vulnerabilities to disease [39].

## Methods

### DNA methylomes

DNA was extracted from peripheral blood of five chimpanzees (*Pan troglodytes*; three males, two females) and five rhesus macaques (*Macaca mulatta*; three males, two females). Samples were taken from captive individuals at Tierpark Nordhorn, Basel Zoo, Leipzig Zoo and at the German Primate Centre during routine health checks and not specifically for this study. Microsatellite analysis conducted at the German Primate Centre verified that respective individuals are not related. Sample collection adhered to the American Society of Primatologists' Principles for the Ethical Treatment of Non-Human Primates [40]. Human DNA was derived from 10 anonymous healthy human subjects (also 60% male).

Analysis was performed in whole peripheral blood in all primates in order to identify significant outlier changes. The proportional makeup of cells present in the blood, which are predominately neutrophils (47 to 67%) and lymphocytes (28 to 37%) in human [32], has very similar ratios in chimpanzee (male neutrophils 59%, lymphocytes 28%; female neutrophils 49%, lymphocytes 41% [33]) and rhesus macaque (neutrophils approximately 67% and lymphocytes 26 to 35% [34]). Whilst additional populations of cells, as well as extra subtypes such as lymphocytes, T cells (CD4, CD8, and others), B cells, natural killer cells, and others, will each possess their unique subtle signatures, this is far below the resolution of this study as only large significant global changes were examined.

DNA samples were pooled for each species at equal concentration for each individual to obtain averaged methylomes and reduce individual genotypic polymorphism effects. Methylated DNA immunoprecipitation (MeDIP was then executed according to the Auto-MeDIP-seq protocol as described in Butcher and Beck [41] and sequenced on an Illumina GAIIx. We generated a data set of over 171 million uniquely mapped fragments (>342 million mapped paired-end reads). Of these, 40,797,356 were human, 64,610,346 were chimpanzee and 65,824,761 macaque. This was performed with paired end reads of 36 bp with average fragment sizes of 197 bp in human, 222 bp in chimpanzee, and 217 bp in macaque.

MeDIP-seq data were processed using MeDUSA v1.0 (Methylated DNA Utility for Sequence Analysis) [42]. Sequence quality control was performed using FASTQC [43]. MeDUSA utilized BWA (v0.5.8) [44] for alignment to reference genomes. Human was aligned to hg19, chimpanzee to panTro2 and macaque to rheMac2 obtained from University of California Santa Cruz (UCSC). Alignment was performed with default parameters. Following alignment a number of filtering steps were performed. Initially SAMtools v0.1.18 [45] was used to remove reads with low alignment score (q < 10). Only those forming a correctly aligned pair were kept. A final filtering step removed potential PCR artifacts by discarding all but one read pair within groups of non-unique fragments. MeDIP specific quality control was performed using MEDIPS (v1.0) [46]. Bigwig files representing normalized read depth (reads per million or rpm) were generated for viewing in the relevant genomes. The corresponding methylome data were deposited in Gene Expression Omnibus (GEO) under accession number GSE48942.

### Differential methylation region analysis

Regions enriched for methylated reads were identified using both MACS v1.4.1 [47] and BayesPeak v1.8 [48]. Both programs were run using the alignment files obtained from their native genome (that is, prior to liftOver). MACS was run using the parameters -nomodel with bandwidth set to the alignment calculated fragment length (human = 197, chimpanzee = 220 and rhesus macaque = 217) and shiftsize to half the fragment length. A *P*-value threshold of 1 × 10^-3^ was used for peak selection. Bayes-Peak was run with default values in multicore mode. Having identified the regions enriched for MeDIP-seq signal, to enable comparative analysis of these peak regions, the MACS and BayesPeak output for chimpanzee and rhesus macaque was lifted to human hg19 using liftOver [49] with minMatch = 0.7. This parameter is appropriate due the recent shared evolutionary history of these species [50]. Subsequently, human-specific differentially methylated regions (s-DMRs) were located through a series of intersection analyses utilizing the BEDTools [51] command intersectBed (v2.10) with a minimum overlap threshold of 1 bp.

Regions hypomethylated in human were defined as regions containing peaks in chimpanzee and rhesus macaque but not in human. Firstly, a single peak set was defined for both chimpanzee and macaque by excluding any MACS peaks that failed to intersect with a Bayes-Peak call. Secondly, shared peaks were identified between the chimpanzee and macaque peak set prior to intersecting with both the human MACS and human BayesPeak set. Hypomethylated s-DMRs were defined by the presence of a shared chimpanzee/rhesus macaque peak in the absence of a human peak from either software output. The chimpanzee MACS peak location lifted to human determined the coordinates of the s-DMR. A similar process was followed to identify hypermethylated s-DMRs in human. Any regions found in both MACS and BayesPeak in human, but that did not intersect any peak in chimpanzee or rhesus macaque, were isolated. Potentially, false positive hypermethylated regions could be generated, caused by an issue in the conversion from non-human primate genome to human genome. To remove such errors, all potential hypermethylated regions were lifted back to their non-human primate genome and then again to human. Only those peaks mapping back to the original location were maintained as hypermethylated s-DMRs for further analysis. We furthermore excluded all of the declared poor mappability regions as identified by ENCODE [52].

To benchmark the robustness of our peak calling method we compared the raw read counts for each species within their native genomes within the lifted s-DMRs coordinates. Normalizing reads between all three of the species to a human constant ((Reads/Total aligned individual species reads) × Total human reads), we showed that there were significant differences in methylation signal prior to liftOver. The comparisons between human and chimpanzee and human and rhesus macaque are significantly greater in the hypermethylated s-DMRs and are significantly less in the hypomethylated s-DMRs, versus the difference between chimpanzee and rhesus macaque in these locations (all human differences versus non-human differences pairwise comparisons; Wilcoxon tests all *P* < 2.2 × 10^-16^; data not shown).

### s-DMR enrichment analysis

Feature enrichment data were visualized using Epiexplorer [53]. Significance was calculated by Genomic Hyperbrowser [54] via hypothesis overlap testing with ChromHMM GM12878 (*P*-values were computed under the null model defined by the following preservation and randomization rules: preserve segments (T2), segment lengths and inter-segment gaps (T1); randomize positions (T1), Monte Carlo (MC) false discovery rate (FDR) threshold 0.005). Gene enrichment via GREAT 2.0.2 Region-Based Binomial Analysis was performed with the default Basal + extension parameters (constitutive 5.0 kb upstream and 1.0 kb downstream, up to 1000.0 kb max extension). Curated regulatory domains were included. For the hypermethylated s-DMR repeat enrichment, the control set was calculated within Epiexplorer [53] by reshuffling the genomic positions while retaining the overall number of regions and the distribution of region sizes.

### CpG islands and shores

CpGi annotation was obtained from Ensembl (build 64). CpGi shores were defined as regions extending 2,000 bp upstream and downstream of each CpGi. The BEDTools [51] function intersectBed, using a minimum overlap of 0.5, was used to determine the number of s-DMRs within each feature.

### Transcription factor binding sites

FASTA sequence for each of the s-DMRs was obtained from the reference sequence for human and the orthologous sequence obtained for chimpanzee and rhesus macaque via LiftOver [49] with the BEDTools [51] FastaFromBed function. The transcription factor affinity prediction tool TRAP [55] (multiple sequences) was implemented with the 904 TRANSFAC [56] motifs (transfac_2010.1 vertebrates), background model of human promoters, and Benjamini-Hochberg multiple test correction. DMRs greater than 5 kb were excluded from this analysis. The SP1 motifs investigated included SP1_01, SP1_Q6, SP1_Q6_01, SP1_Q4_01, SP1_Q2_01 and SP1_02; and the RFX family members included RFX1_01, RFX1_02, RFX_Q6 and EFC_Q6. The empirical *P*-value calculation for CTCF was derived by randomization with R (4 by 1,000×). A set of random hypomethylated and hypermethylated s-DMRs, from the total set of shared chimpanzee and rhesus macaque peaks, and all human peaks, respectively, that overlap CpGis were selected and TRAP [55] motif prediction scores were calculated for CTCF_01 and CTCF_02 in the human as well as orthologous chimpanzee and rhesus macaque sequences (via LiftOver and FastaFromBed). Those results that exceeded the observed human CTCF motif divergence were then calculated.

### Exons and gene body methylation

Hypomethylated sDMRs located in CpGis were further filtered to obtain a subset associated with 5′ promoter regions. The exons associated with the selected promoters were identified and their methylation status was compared in order to detect inverse changes in gene body methylation. This was performed by identifying MACS peak regions in the human data within the exons and comparing the MACS score (the transformed *P*-value attributed to the peak) between species. If no peak was found within an exon for either chimpanzee or rhesus macaque, a score of 0 was given. The resulting regions were ranked according to differential MACS score.

### Statistical analysis

All other statistical analyses were performed in the R environment [57]. Chi-squared calculations for enrichment for hypomethylated s-DMRs, were compared with the combined chimpanzee and rhesus macaque peak set (94,799 peaks) and those for hypermethylated s-DMRs, with the total human peak set (133,494). For the genomic feature intersection calculations, the Bayes Peak confirmed MACS peak coordinates were used.

### Additional epigenomic datasets

Neutrophil MethylSeq (digestion with HpaII followed by sequencing) data from Martin *et al*. [19] for four humans and four chimpanzees was accessed from GEO accession number GSE22376. Results were expressed as the probability p(U) that the site is unmethylated, and vary between 0 (methylated site) and 1 (unmethylated site). The probability of being methylated was then taken as 1 - p(U), with 0 = unmethylated and 1 = methylated. Values across the two human CpGis and chimpanzee CpGis were averaged across the island. Comparative H3K4me3 ChIP-seq data from Cain *et al*. [21] were accessed via GEO (GSE24111). These data were derived from B cells (lymphoblastoid cell line) from three humans, three chimpanzees and three rhesus macaques.

### Functional analysis of Leukotriene B4 Receptor

#### Materials

LTB_4_ and LY293111 were purchased from Cayman Chemical (Ann Arbor, MI, USA). Calcium ionophore (A23187) was purchased from Sigma-Aldrich (Dorset, UK). All other materials were purchased from Life Technologies (Carlsbad, CA, USA) unless otherwise stated.

#### Isolation of peripheral blood mononuclear cells

Isolation of human PBMCs was performed by density centrifugation using Polymorphprep (Axis-Shield, Oslo, Norway) according to the manufacturer’s protocol. The study was approved by the Research Ethics Committee of Guy’s Hospital REC (no: 09/H804/077) and participants had provided written consent prior to any procedure. In brief, blood collected over 5.4 mmol/L EDTA was layered over an equivalent volume of Polymorphprep and centrifuged (500 g) for 35 minutes at 20°C. PBMCs collected were resuspended in 1:1 volume of RPMI:H_2_O to restore osmolarity. After incubation with RBC lysis buffer (eBiosciences, San Diego, CA, USA) to eliminate erythrocyte contamination, cells were then washed twice in phosphate-buffered saline and counted using a NucleoCounter (Chemometec, Allerød, Denmark). Isolation of rhesus macaque PBMCs was performed at the German Primate Centre by standard protocols for human PBMCs using Biocoll 1.077 g/ml (Biochrome, Cambridge, UK) with an extended centrifugation step (45 minutes) for separation of PBMCs. Separate PMBC aliquots in Trizol and in medium (HEPES-buffered RPMI + 10% fetal calf serum + Pen/Strep) were transported.

#### Real-time PCR

Total RNA was extracted from cells lysed in Trizol (Life Technologies) using QIAshredder columns and a RNeasy mini kit (QIAGEN, Venlo, Limburg, Netherlands) with TURBO DNase (Ambion, Life technologies, Carlsbad, CA, USA) treatment following the manufacturer’s protocol. RNA was quantified using a NanoDrop ND 1000 spectrophotometer (Thermo Scientific, Waltham, MA, USA) and ND-1000 software version 3.2.0. Reverse transcription of RNA was carried out using RevertAid M-MuLV reverse transcriptase (Fermentas, Thermo Scientific, Waltham, MA, USA) and primed using random hexamers according to the manufacturer’s protocol. Relative mRNA expression levels were measured using TaqMan Gene Expression Master Mix (Applied Biosystems, Life technologies, Carlsbad, CA, USA) and the following TaqMan probe sets: LTB4R- Hs01938704_s1 and 18s RNA- 4319413E, matching both human and rhesus macaque sequences. RT-PCR was performed on a ViiA 7 Real-Time PCR system (Applied Biosystems) and analyzed using ViiA 7 software version 1.0.

#### Calcium mobilization assay

Calcium mobilization assays were conducted using a FLIPR calcium 4 assay kit (Molecular Devices, Sunnydale, CA, USA) as described previously [58,59]. PBMCs (2 × 10^5^/well) were plated into poly-D-lysine-coated 96-well plates in RPMI 1640 supplemented with 10 mmol/L HEPES. Cells were incubated for 1 hour with FLIPR loading buffer prior to addition of ligand and fluorescent intensity was measured at 37°C using a Flexstation 3 (Molecular Devices). Results were analyzed with SoftMax Pro Software (Molecular Devices).

### Ethics

Chimpanzee and rhesus macaque samples were taken from captive individuals at Tierpark Nordhorn, Basel Zoo, Leipzig Zoo and at the German Primate Centre during routine health checks and not specifically for this study. Sample collection adhered to the American Society of Primatologists' Principles for the Ethical Treatment of Non-Human Primates [40]. For the isolation of human PBMC samples for functional analysis, the study was approved by the Research Ethics Committee of Guy’s Hospital (REC no: 09/H804/077) and participants had provided written consent prior to any procedure.

## Results

### Primate DNA methylomes

The DNA methylomes of peripheral blood DNA samples were generated from pooled individuals in order to reduce the effects of individual genetic variability. These were of healthy unrelated primates, comprising 10 humans, 5 chimpanzees and 5 rhesus macaques, all with a 60% male split. Also, uncultured cells were used to avoid the incorporation of additional stochastic artifacts [60] Automated MeDIP-seq was performed as previously described [41], and data were processed using the MeDUSA pipeline [42].

The DNA methylomes are displayable in the individual genomes, human (GRCh37), chimpanzee (CGCS 2.1/PanTrog2) and rhesus macaque (MGSC Merged 1.0/rheMac2) in the UCSC genome browser in the context of existing annotation and are available at [61]. The chimpanzee and macaque results can also be viewed on the human sequence having been converted using the liftOver utility [49] to enable an initial comparative view.

### Human species-specific differentially methylated regions

To find loci with human s-DMRs we first identified robust enrichment peaks using the MACS v1.4.1 [47] peak-calling algorithm, but with the additional conservative requirement that regions must also have an overlap with a BayesPeak v1.8 [48] algorithm result (see Methods). This additional step reduced the number of potential peaks by up to approximately 8%. We then identified all human peaks where no chimpanzee or rhesus macaque peak was present in the orthologous location (with either algorithm) to define hypermethylated human s-DMRs, and hypomethylated human s-DMRs where no human peak was present (again with either algorithm) but where both chimpanzee and macaque peaks were located in common.

**Figure 1:**
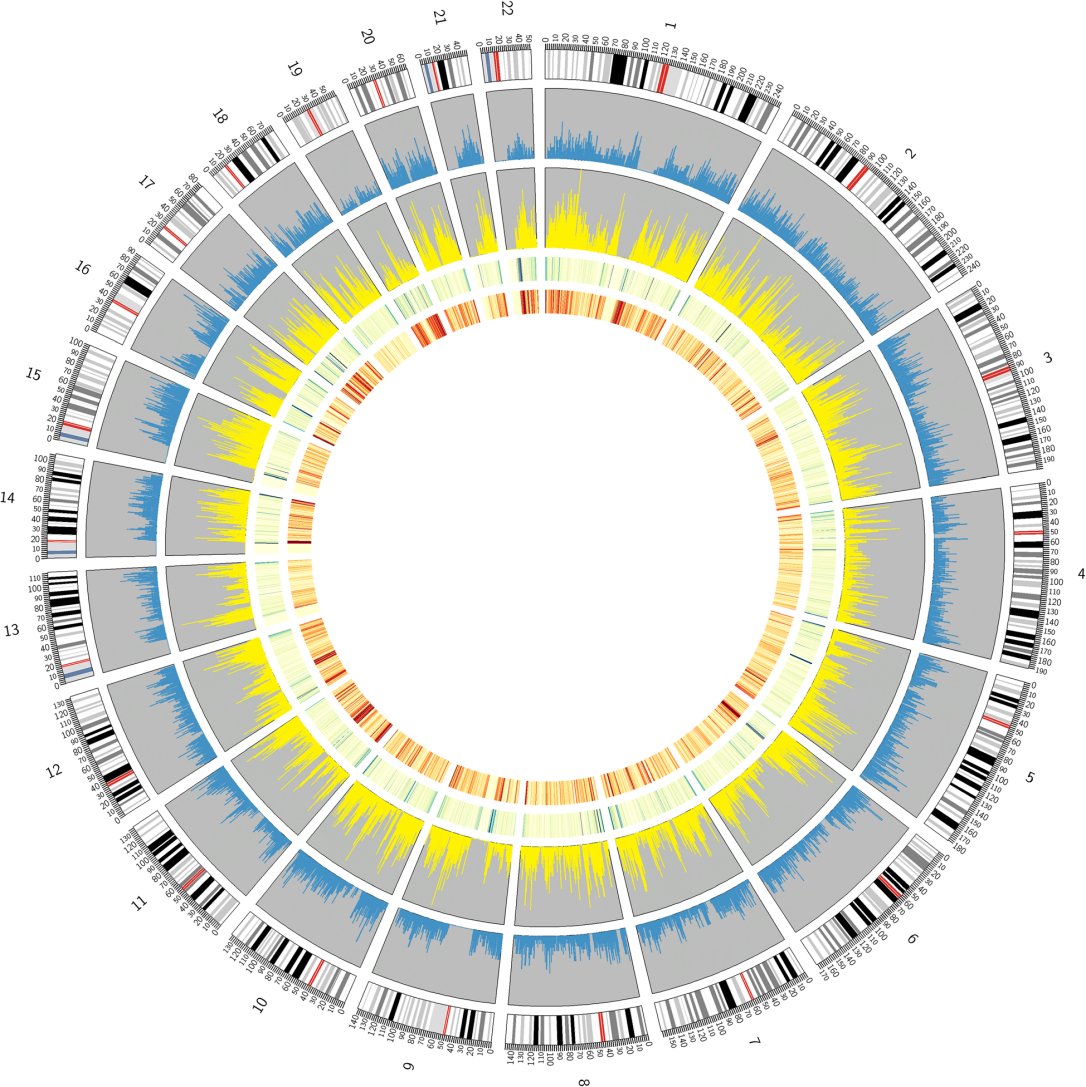
**Genome-wide view of human s-DMRs.** Pooled DNA from uncultured whole blood cell samples including both sexes (all 60% male) were analyzed for each species. Methylated fragments via MeDIP-seq were aligned to the appropriate species’ genome; then peaks were called within these, using two peak-calling algorithms (MACS v1.4.1 [47] and BayesPeak v1.8 [48]). Triangulation reciprocal LiftOver [49] comparison then identified human s-DMRs. Outer ring, human chromosomes; blue ring, hypermethylated human s-DMRs (15,858); yellow ring, hypomethylated human s-DMRs (22,758); green ring, CNV density; inner ring, gene density.

A total of 22,758 hypomethylated and 15,858 hypermethylated human s-DMRs were identified. All of these lie in strongly sequence-similar regions between the three primate genomes, but are also unique within each primate genome due to the requirement of these s-DMRs to be able to pass through consistent reciprocal liftOver [49] steps across the primate genomes. Therefore, whilst genetic influence on the methylome is strong and high sequence similarity exists, particularly between human and chimpanzee (approximately 98.6% [62]) and primates overall [63,64], substantial numbers of s-DMRs were identified. Without the additional out-group of rhesus macaque the calculated human-specific set via only a comparison with chimpanzee would be 69.2% larger; thus, its inclusion increases our power to identify true human-specific modification. The number of hypomethylated human s-DMRs is moderately greater compared with hypermethylated s-DMRs, which is likely due to an increased loss within the human peak set, as these must pass through reciprocal liftOver to the two slightly less well-characterized genome sequences of chimpanzee and macaque. Consistent with this, peaks from human chromosome 21 lifted via the higher quality chimpanzee chromosome 21 [65] retained the highest proportion of peaks of any chromosome (data not shown). These regions are visible as hypomethylated (yellow) and hypermethylated (blue) s-DMRs also via the above UCSC tracks link and are visualized genome-wide in Figure 1[66].

### Genome feature annotation enrichment

**Figure 2:**
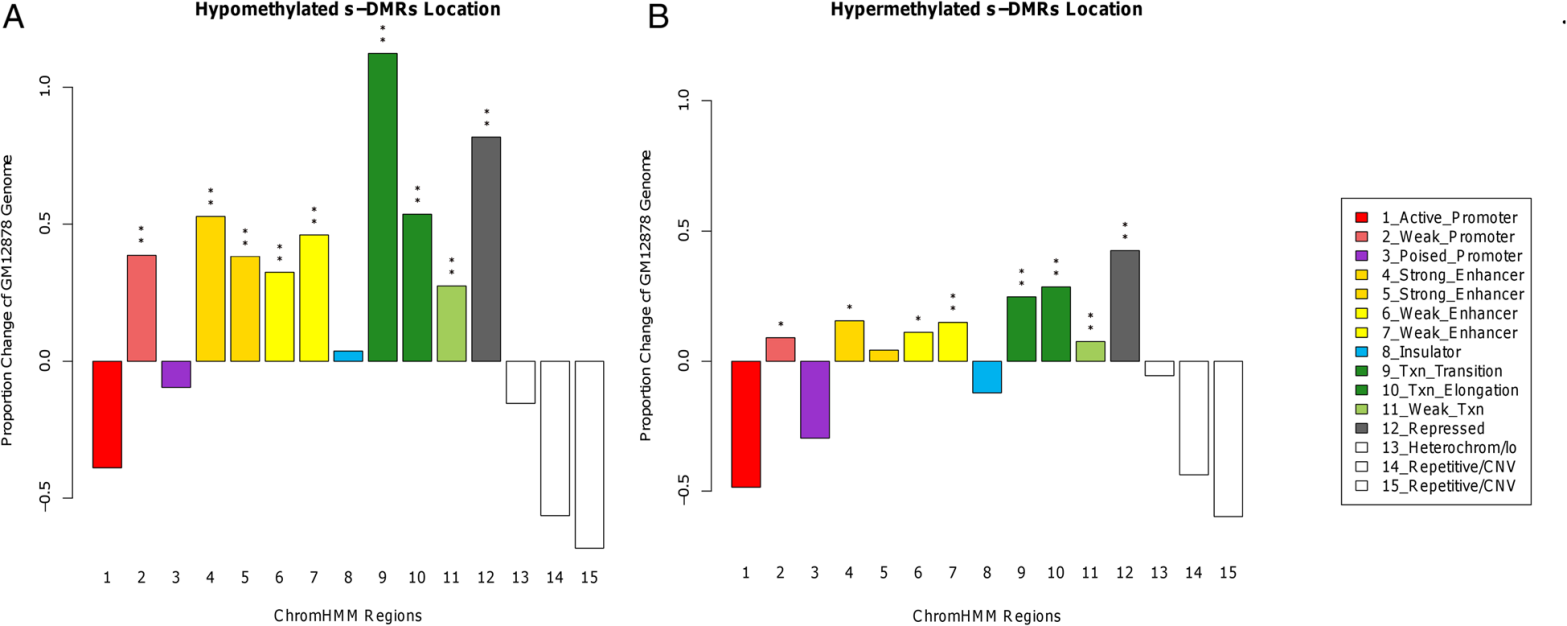
**Proportional genomic annotation coverage of s-DMRs compared with HapMap B-lymphocyte EBV GM12878 ChromHMM data**[68]**. (A)** Hypomethylated s-DMRs; **(B)** hypermethylated s-DMRs. Various genomic annotations were significantly enriched, as defined by this segmentation analysis, calculated via the Genomic Hyperbrowser [54] (*P*-value overlap MC * < 0.05, ** < 0.005).

We compared the location of human s-DMRs to genomic functional annotation, as defined by the ChromHMM segmentation analysis [67], using the HapMap line interrogated by Encode (B-lymphocyte GM12878 data). We calculated significance via Epiexplorer [53] and the Genomic Hyperbrowser [54]. This showed a similar pattern in both hypo- and hypermethylated s-DMRs, with a significant skew towards weak promoters, gene transcriptional regions, and both strong and weak enhancers, but depletion within strong promoters (Figure 2A,B). This is comparable to the finding in tissue and reprogramming s-DMRs, with most DMRs in moderate CpG dense regions [36]. Increased levels of s-DMRs in these transcription-associated regions may be indicative of the potential role methylation variability plays in gene body methylation [68] and splicing [69,70]. The number of hypomethylated s-DMRs residing over exons is 3,444 and over introns is 9,848, and for hypermethylated s-DMRs these numbers are 1,042 for exons and 6,514 for introns. The more consistent and stronger results of hypomethylated s-DMRs in enhancer regions is consistent with studies showing these regions may be protected from DNA methylation [71,72] and are more species-specific. Furthermore s-DMR genomic regions are enriched in comparison to the rest of the genome for the dynamic fraction of the DNA methylome as defined by Ziller *et al*. [73] (data not shown, χ^2^*P* < 2.2 × 10^-16^).

### Gene enrichment

Gene enrichment analysis of these s-DMR regions was performed using the Genomic Regions Enrichment of Annotations Tool (GREAT) [74]. A large number of significant biological, human phenotype and disease-related enrichments were identified by FDR-corrected region-based binomial analysis (Additional file 1). A smaller set of intriguing categories were found to be significant in both the hypo- and hypermethylated s-DMR enrichment categories. This included monocyte activation and regulation of prostaglandin-endoperoxide synthase activity for biological processes; hypotrichosis for human phenotype; and diverticulitis for disease ontology, which has not been observed in non-human primates to date [75]*.* The evolutionary timescale difference between human and chimpanzee divergence (5 to 6 MYA) and human population-based polymorphisms (<1 MYA) is large. Therefore, many potential genetic disease susceptibilities will be fixed between the species. Nevertheless genome-wide association study SNPs [76] are enriched in these s-DMR regions by approximately 1.40× compared to the genome average (data not shown; χ^2^*P* = 5.09 × 10^-3^).

### Repetitive elements

**Figure 3:**
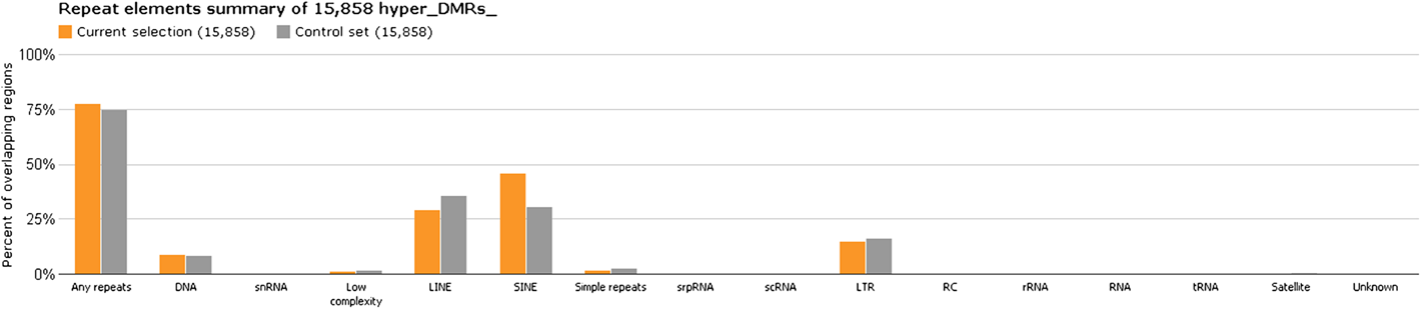
**Subcategorization of repeat element increase in hypermethylated s-DMRs via Epiexplorer in comparison with a reshuffled control set**[53]**(medium overlap ≥10%).** Increased hyper s-DMRs within the SINE group are identified, which comprises predominately *Alu*s. LINE, long interspersed nucleotide element; LTR, long terminal repeat; RC, rolling circle; snRNA, short nuclear RNA; srpRNA, signal recognition particle RNA.

The hypermethylated s-DMR set was found to be increased within repetitive elements, specifically the SINE group (Figure 3; via Epiexplorer [53]). Examination of this result showed that these hypermethylated s-DMRs were disproportionally increased within the second oldest *AluS* subcategory (χ^2^*P* < 2.2 × 10^-16^), which still possesses mobilization ability [77], whilst being proportionally reduced within both the most ancient *AluJ* and youngest *AluY* categories (Additional file 2). Moreover, hypomethylated s-DMRs also showed this pattern, with an increase in *AluS* (χ^2^*P* < 2.2 × 10^-16^), but also showed a slight increase in the youngest and most active *AluY* (χ^2^*P* = 1.15 × 10^-4^). Transposable elements constitute nearly half of the primate genome and there is increasing evidence for their functional role in influencing expression and potential modulation in human pathology [78]. Primate-specific open chromatin regions (DNAse I hypersensitivity sites) are enriched for transposable elements (approximately 63%) [79] and methylation changes in *trans*-species experiments have also indicated their regulatory potential [80].

### CpG islands

#### s-DMRs are more prevalent in CpG shores than CpG islands

**Figure 4:**
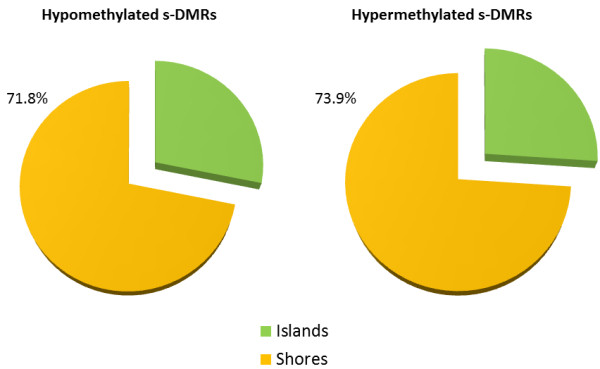
**Human s-DMRs in CpG islands versus CpG island shores.** Number of hypo- and hypermethylated s-DMRs for island (77 and 45) and shore (821 and 431), respectively, is corrected for proportion of genome size (genomic space for islands = 23.8 Mb and for shores = 89.5 Mb). Directly comparing island and shore s-DMRs with regard to possible locations of hypomethylated s-DMRs (all co-locating chimpanzee and rhesus macaque peaks in these regions), χ^2^*P* = 2.90 × 10^-29^, and hypermethylated s-DMRs (from all location of human peaks in these regions), χ^2^*P* = 1.93 × 10^-8^; combined χ^2^*P* = 1.80 × 10^-32^. Therefore, human-specific peaks are more likely than non-human-specific peaks to reside within CpGi shores.

Previously identified tissue-, cancer- and reprogramming-specific DMRs [81,82] have all been found to be more prevalent in moderate CpG dense regions surrounding CpGis, termed CpGi ‘shores’, than within the island themselves. Within the CpGis and CpGi shores, we identified 77 hypo- and 45 hypermethylated human s-DMRs, and 821 hypo- and 431 hypermethylated s-DMRs, respectively (Additional file 3). After accounting for the almost fourfold larger genome size taken up by shores (approximately 89.5 Mb) than islands (approximately 23.8 Mb), these s-DMRs were still more prevalent within shore regions (Figure 4; Wilcoxon test, both hypo- and hypermethylated, *P* < 2.2 × 10^-16^). Additionally, this was also significant when comparing island and shore s-DMRs with regard to all possible locations of hypomethylated s-DMRs (all co-locating chimpanzee and macaque peaks in these regions; χ^2^*P* = 4.80 × 10^-29^) and hypermethylated s-DMRs (from location of all human peaks in these regions; χ^2^*P* = 2.74 × 10^-8^) and for s-DMRs combined (χ^2^*P* = 2.94 × 10^-32^). Therefore, the proportion of s-DMRs in CpGi shores is far higher than in islands (14.0 compared to 5.13 per Mb of feature sequence). However, other features are even more concentrated with s-DMRs: exons (23.7/Mb), CTCF sites (26.8/Mb) and DNAse I hypersensitivity sites (40.3/Mb).

#### Sequence similarity of s-DMRs in CpG islands

**Figure 5:**
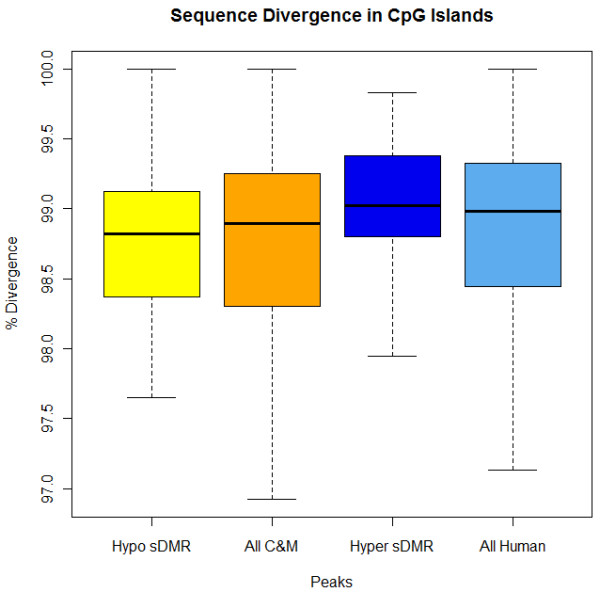
**Sequence divergence between human and chimpanzee within peak regions.** There was no significant difference (Wilcoxon *P* > 0.05 for both) between s-DMRs in islands and all chimpanzee and rhesus macaque (C&M) combined peaks or all human peaks in islands.

Sequence divergence could be contributing to the s-DMRs within these CpGis [27]. Therefore, to assess whether there was greater divergence between species within s-DMRs, we compared the sequence similarity between the s-DMRs and the total background set of peaks that these were identified from. However, no significant difference in sequence change between human and chimpanzee was seen between hypomethylated s-DMR peaks and the total combined non-primate peaks, or hypermethylated s-DMR peaks and the total set of human peaks within CpGis (Wilcoxon test, both non-significant, *P* > 0.05; Figure 5). The observed sequence divergence for all four subsets is at the expected global genomic level of approximately 99% sequence identity. This does not rule out sequence influence within these s-DMRs, but implies that more severe genetic differences would not have passed our reciprocal liftOver requirements and within these s-DMR regions sequence change is not more prevalent than the genome average. Therefore, only minor levels of base change may be contributing to this methylation variation.

#### Transcription factor binding site modification

Sequence alteration in certain TFBS motifs within CpGis have been shown to modify local methylation and thereby act as MDRs [27]. With the availability of both human s-DMRs from our dataset and sequence data from these three species, we were able to interrogate these regions for potential MDRs. Isolated species-specific genetic changes significantly modifying TFBS motif strength were looked for. To do this we utilized the Transcription Factor Affinity Prediction (TRAP) tool [55] and calculated the binding prediction of 904 TFBSs (TRANSFAC vertebrate V$motifs [56]) within CpGi-related s-DMRs in human, as well as the orthologous chimpanzee and macaque sequences.

**Figure 6:**
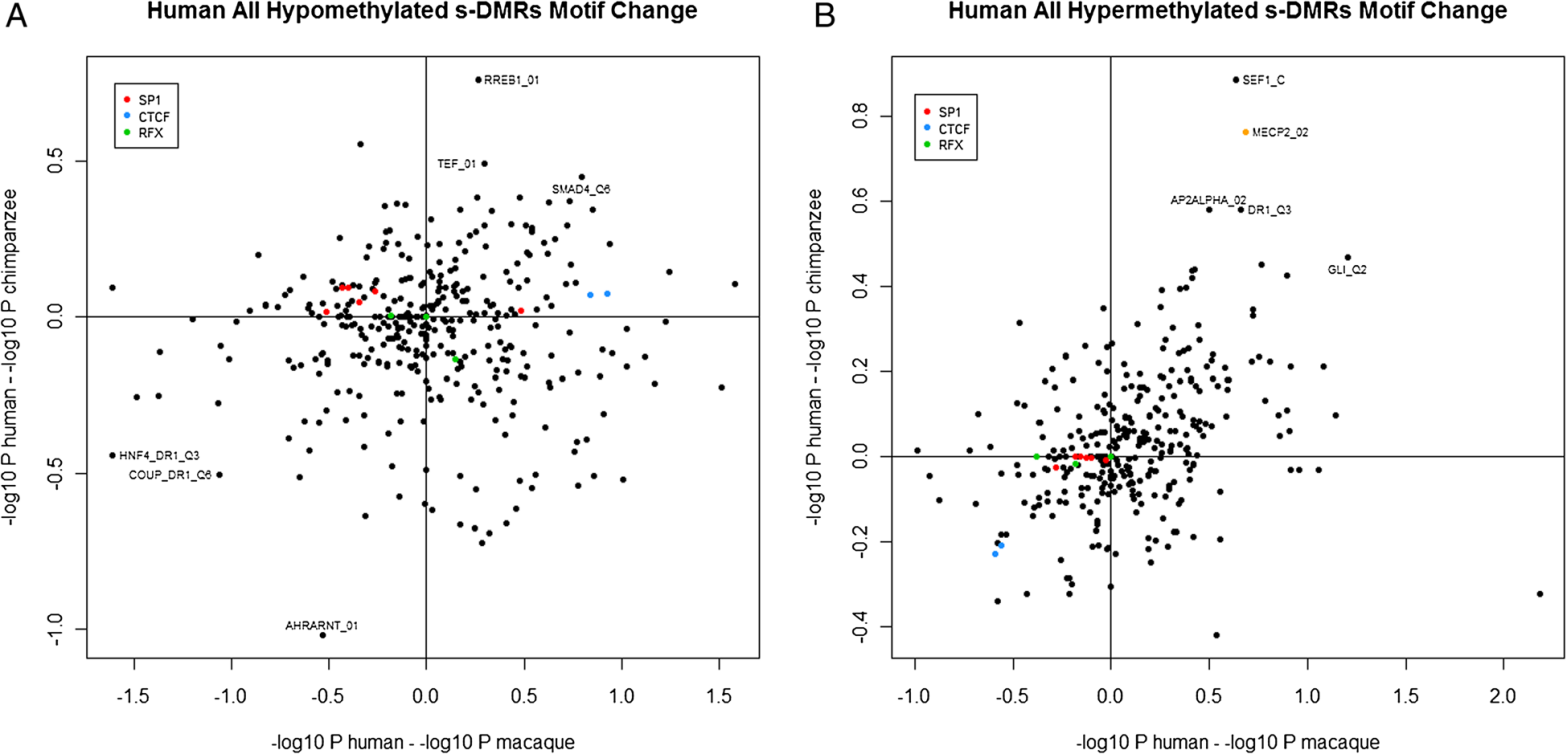
**Change in transcription factor motif binding prediction within s-DMRs between primates calculated via TRAP [**[55]**] with TRANSFAC motifs**[56]**. (A,B)** Difference in binding prediction (total corrected Benjamini-Hochberg -log_10_*P*-value) between human and chimpanzee (y-axis) and human and rhesus macaque (x-axis) for each motif within the total set of hypomethylated **(A)** and hypermethylated **(B)** CpGi s-DMRs. Known TFBSs with MDR effects are highlighted in color (SP1 in red, CTCF in blue, RFX motif family in green). Both CTCF motifs show a consistent increase in the hypomethylated s-DMRs, as well as a consistent decrease in the hypermethylated DMRs, with respect to human. The MeCP2 motif is identified as a strongly increased outlier in the hypermethylated s-DMRs (orange).

We performed a global comparison for the total set of hypomethylated and hypermethylated CpGi s-DMRs by calculating a single total binding *P*-value (Benjamini-Hochberg corrected) for each motif in each of the three species. This could be performed in these CpG dense, predominately hypomethylated and classical promoter regions as a slight MDR motif sequence variation would be detectable in these highly similar orthologous sequences. We visualized this by plotting in two dimensions the difference in *P*-values (-log_10_) between human and chimpanzee (on the y-axis) and human and macaque (on the x-axis) binding scores to identify consistent human directional change (Figure 6A,B). Both CTCF motifs (CTCF_01 and CTCF_02) showed consistently increased and decreased motif binding in human hypomethylated and hypermethylated s-DMRs, respectively (blue dots in Figure 6A,B; Additional file 4; empirical *P* = 0.046). That is, there was an increase in human CTCF binding compared to both chimpanzee and macaque within hypomethylated s-DMRs, and inversely a consistent decrease in CTCF binding in hypermethylated s-DMRs. The CTCF motif has been previously identified as an MDR [27]. This *trans*-species effect is consistent with the disruption of CTCF occupancy being linked to increased methylation, recently observed across numerous human cell types [83], as well as the different role of CTCF binding regions within open and closed chromatin [84]. However, other potential MDRs, such as SP1 motifs [85,86] and members of the RFX family, did not show consistent human directional divergence in this global analysis. One of the most extreme outliers within the hypermethylated set was for increased human binding strength of a MeCP2 (MECP2_02) motif. Due to the critical role of this gene in development and neural function [87,88], this may represent motif variation that is contributing genetically to epigenetic effects across multiple tissues.

#### Canonical 5′ promoter s-DMRs

From the total s-DMRs within CpGis, a smaller subset of 53 hypomethylated and 25 hypermethylated s-DMRs reside within the canonical 5′ promoter region, and as such would have a recognized strong potential association with the expression of these genes [89].

**Figure 7:**
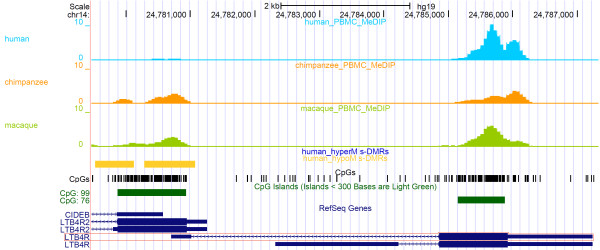
**Comparative DNA methylation of *LTB4R *visualized in the UCSC browser. Human hypomethylated s-DMRs (yellow) are shown in the promoter CpGi (CpG: 99) of *LTB4R *(major isoform LTB4R-001 outlined in red).** Methylation scale is in reads per millions (RPM) for each species from MeDIP-seq (human, light blue; chimpanzee, orange; rhesus macaque, olive green). As well as reduced promoter methylation, larger gene-body methylation, which is related to higher expression [69], was also seen in human compared with the other species over the sole exon (approximately 1.29-fold stronger peak MAC *P*-value over gene body CpGi (CpG:76)). In this complex locus the promoter of the major isoform of *LTB4R* (highlighted with a red rectangle) also co-locates with the gene body of the low-specificity receptor *LTB4R2* and *CIDEB. LTB4R* has strong expression in all blood subtypes, particularly the myeloid lineage, including monocytes (Additional file 6).

As highly expressed genes are associated with low gene promoter methylation and high gene body methylation [90], we ranked these loci by differential gene body methylation between human and averaged chimpanzee and rhesus macaque methylation levels in the consensus transcripts (CCDC) that arise from these promoters (Table S4A in Additional file 5). The top hypomethylated promoter with greatest inverse differential in gene body methylation was the major isoform of *LTB4R* (Leukotriene B4 Receptor; Figure 7). This 5′ CpGi in fact also possesses two hypomethylated s-DMRs, with one also overlapping the CpGi shore region. This promoter also overlaps the Leukotriene B4 receptor 2 gene (*LTB4R2*) [91] and is a bidirectional promoter for *CIDEB* transcribed in the reverse direction. As mentioned, the major isoform of *LTB4R* showed higher exonic methylation than both chimpanzee and rhesus macaque (MACS -log_10_*P*-value 2943.7, versus 2397.8 and 2180.8) than any of the other 5′ CpGi hypomethylated genes, with these other co-locating genes (*LTB4R2* and *CIDEB*) showing the inverse result (Table 1). We excluded any build inconsistencies influencing this result by also re-aligning to additional available primate builds (CSAC 2.1.4/PanTrog4 and BGI CR_1.0/rheMac3) and these all showed the identical comparative methylation difference and peak calls within this *LTB4R* locus. The gene body methylation analysis within the set of hypermethylated promoter s-DMRs revealed the genes *GIT1*, *ABCG4* and *RUSC2* as the highest ranked. However, in this analysis there was not one strong outlier identified, as there was for the *LTB4R* result (Table S4B in Additional file 5).

**Table 1:** Hypomethylated 5′ human promoter with primate differential exon methylation

**Order**	**Gene**	**Ensembl transcript ID**	**CCDS ID**	**HGNC transcript name**	**Human**	**Chimpanzee**	**Rhesus macaque**	**Human - chimpanzee**	**Human-macaca**	**Human- average**
1	*LTB4R*	ENST00000345363	CCDS9626	LTB4R-001	2,943.7	2,397.8	2,180.8	546.0	763.0	654.5
63	*CIDEB*	ENST00000258807	CCDS32056	CIDEB-001	47.6	205.6	155.5	-158.0	-107.8	-132.9
64	*CIDEB*	ENST00000541830	CCDS32056	CIDEB-201	47.6	205.6	155.5	-158.0	-107.8	-132.9
71	*CIDEB*	ENST00000336557	CCDS32056	CIDEB-002	83.4	374.1	391.4	-290.8	-308.0	-299.4
73	*LTB4R2*	ENST00000533293	CCDS9625	LTB4R2-002	0.0	456.6	477.1	-456.6	-477.1	-466.8
74	*LTB4R2*	ENST00000543919	CCDS9625	LTB4R2-201	0.0	456.6	477.1	-456.6	-477.1	-466.8

Methylation scores from MACS -log_10_*P*-values. *LTB4R* is the most significant. The other co-locating genes, *LTB4R2* and *CIDEB*, rank at the bottom of this set.

### Leukotriene B4 receptor

**Figure 8:**
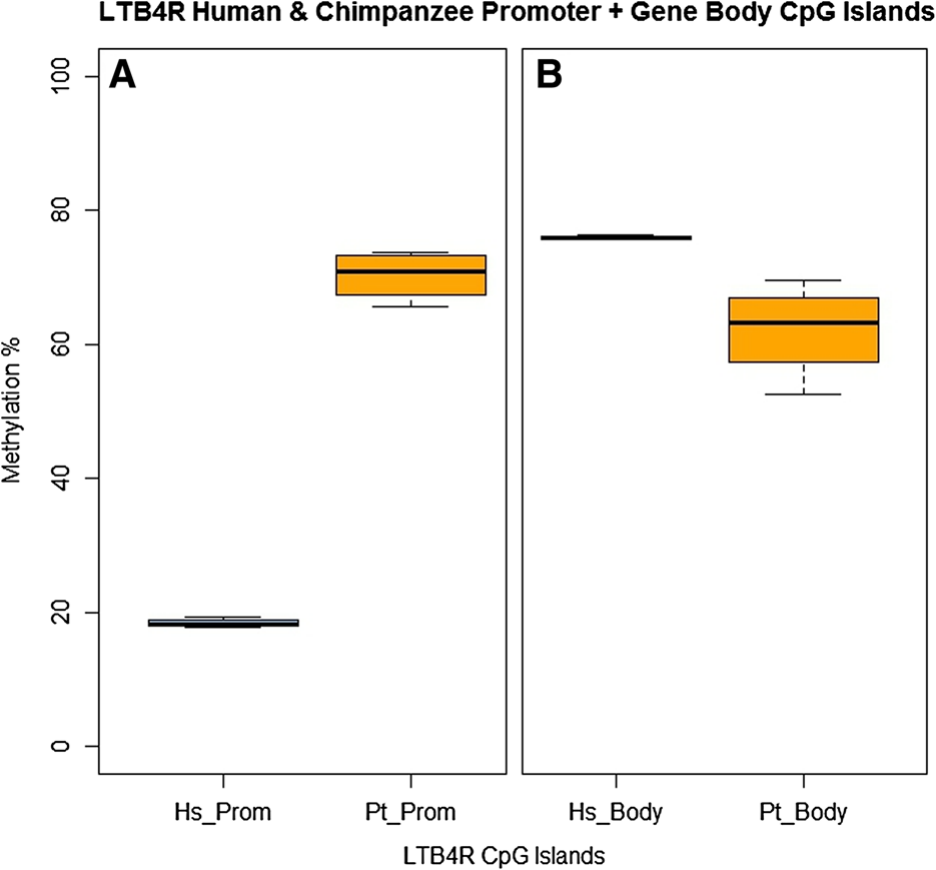
**Results of HpaII digestion of *LTB4R *followed by sequencing for promoter and gene body CpGs in human (Hs) and chimpanzee (Pt) (Martin *et al*.**[19]**via GEO).** Methylation within the **(A)** promoter (Prom = CpG:99 in Figure 7) and **(B)** gene body (Body = CpG:76 in Figure 7) CpG islands of *LTB4R* from reverse scores (1 - p(U)) of all included HpaII MethylSeq sites analyzed by MetMap6 in purified neutrophils. These data replicate the significant difference identified in MeDIP peripheral blood. Four human and four chimpanzee samples had average methylation of 18.4% and 70.3% in the promoter (Prom), and 75.9% and 62.0% in the exonic gene body (Body) orthologous CpGis (Wilcoxon *P* < 0.05 for both).

To investigate the strong comparative methylation difference we had identified in *LTB4R*, we examined the available CpGi-focused methylation-sensitive sequence in neutrophils from Martin *et al*. [19] for four human and four chimpanzees. This replicated these findings within the promoter and exonic CpGis with average methylation of 18.4% and 70.3% across the orthologous CpGi promoter of *LTB4R*, and 75.9% and 62.0% for the exonic CpGi in human and chimpanzee, respectively (Figure 8; Wilcoxon *P* <0.05 for both). This >50% difference in promoter methylation is a level of change only seen within humans in cancer tissue studies, as opposed to the very small variations identified to date in common non-malignant diseases. We also investigated the available human peripheral blood bisulfite-seq DNA methylome from Li *et al*. [92], which also supports the findings in human with a methylation level of 3.43% within the promoter CpGi and 82.22% in the gene-body exonic CpGi.

**Figure 9:**
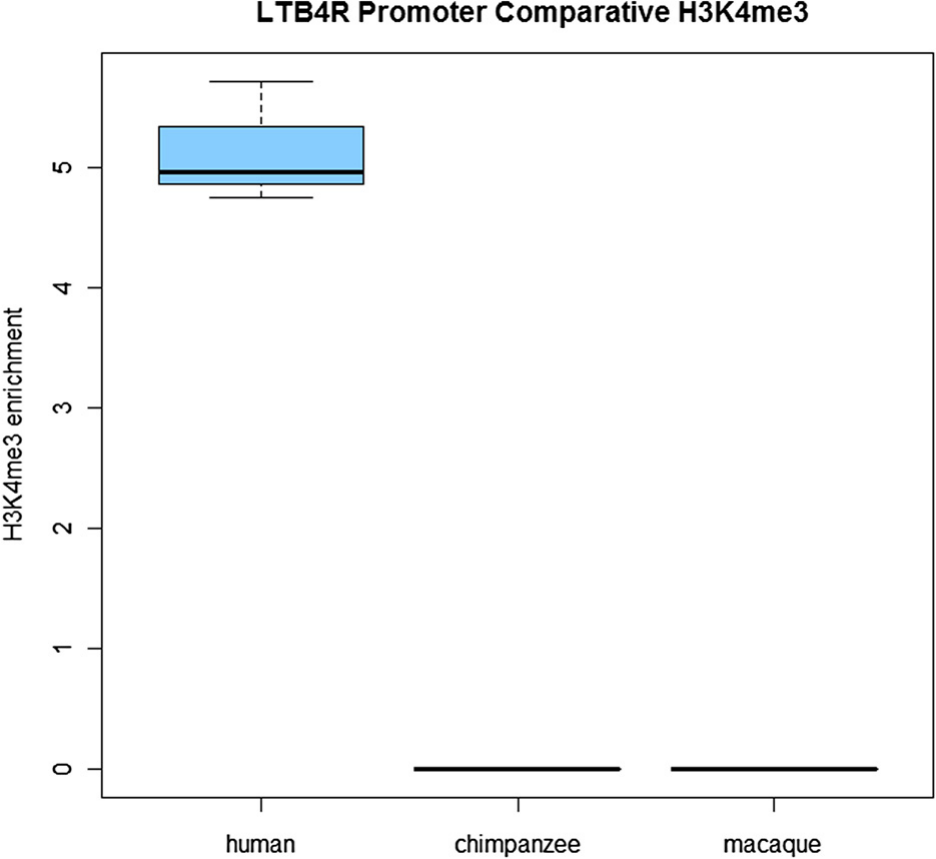
**Human-specific H3K4me3 enrichment in the *LTB4R *promoter from ChIP-seq data derived from B cells (lymphoblastoid cell line; data from Cain *et al. *[**[21]**] via GEO).** These data identified significant activating peaks in all three human samples, but the signal was not strong enough for any peaks to be called in all three chimpanzee and all three rhesus macaque samples analyzed.

Primate comparative chromatin data were available from B-cell lymphoblastoid cell lines [21]. This identified a significant activating H3K4me3 peak in the *LTB4R* promoter of three human samples, but no declared peak in three chimpanzees or three rhesus macaque samples (Figure 9). Thus, although the initial findings were identified in mixed peripheral blood, the strong effect is corroborated in two major blood components; neutrophils and B lymphocyte-derived.

Therefore, these results in individual cell types support that variation in cell type composition is not a potential confounding factor in this DNA methylome analysis from whole blood-derived DNA. However, we further excluded this by examining the 500 leukocyte subtype-related differentially methylated CpG positions (L-DMPs) identified by Houseman *et al*. [93]. We find that none of these CpGs overlap with the entire *LTB4R* locus. Furthermore, just 5 of our 15,858 hypermethylated DMRs (0.032%) and 6 of our 22,758 hypomethylated DMRs (0.026%) show overlap with these L-DMPs, respectively. Therefore, only 11 or approximately 2.2% of these 500 L-DMPs co-localize with any of the s-DMRs identified, indicating that our results are not significantly enriched for cell type-associated methylation changes.

**Figure 10:**
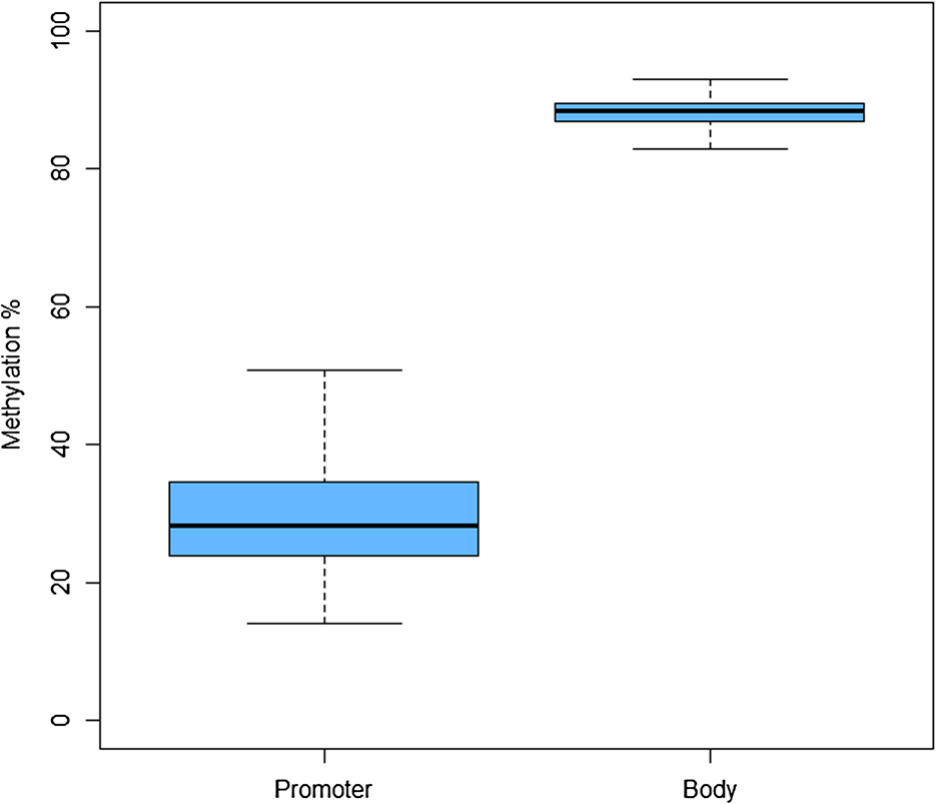
**Data from the MARMAL-AID Human 450k Methylation array repository**[94]**for the *LTB4R *promoter and gene body CpG islands.** These data are derived from 1,665 whole blood samples from healthy and non-cancer disease subjects from multiple experiments. This also showed a consistent low average level of human *LTB4R* promoter methylation (mean 29.4%, standard deviation 7.4%) and high average *LTB4R* gene body methylation (mean 87.9%, standard deviation 2.3%). Human LTB4R methylation whole blood 1,665 samples.

Furthermore, we examined intra-human variability, via the MARMAL-AID repository [94] of all performed 450k DNA methylation array experiments, which includes 1,665 whole blood samples from healthy and non-cancer disease subjects. These data, even derived from multiple studies with significant experimental/batch variation, also confirm consistently low human *LTB4R* promoter methylation (mean 29.4%, standard deviation 7.4% and high *LTB4R* gene body methylation (mean 87.9%, standard deviation 2.3%) (Figure 10).

Methylation of this *LTB4R* promoter has previously been established by Kato *et al*. [95] to reduce this gene's expression. LTB4R (aka BLT1 receptor) is a high affinity G-protein-coupled receptor for LTB_4_, a potent chemoattractant involved in inflammation and immune response in the eicosanoid signaling (leukotriene and prostaglandin) pathway. It has been implicated in the whole spectrum of inflammatory diseases [96], including asthma [97], inflammatory arthritis [98], atherosclerosis [99,100], inflammatory bowel disease [101], and psoriasis [102]. It has also previously been postulated to play a role in HIV infection [103]. *LTB4R* is highly expressed across all blood cell types, particularly in myeloid cells such as neutrophils and monocytes (Additional file 6). The LTB_4_-LTB4R axis is involved in linking early immune system activation, neutrophil auto-signaling and swarming [104], and early effector T-cell recruitment [105], acting as a potent non-chemokine pathway for cytotoxic effector cell traffic [106]. Interestingly, the Gene Ontology category for the ligand of this receptor, ‘Leukotriene Production involved in Inflammatory Response’ (GO:0002540), was significantly enriched in the GREAT analysis for hypomethylated s-DMRs (*P* = 2.76 × 10^-5^, FDR *q* = 2.77 × 10^-4^). This is due to s-DMRs associated with genes such as *ALOX5* (*5-LO*) and *ALOX5AP* (*FLAP*) within this pathway. Furthermore, the gene encoding LTA4 hydrolase (*LTA4H*), which catalyzes the conversion of LTA4 to LTB4, also contains two intragenic s-DMRs.

Whilst CpG density is inversely associated with CpGi methylation state [28,29], the human *LTB4R* promoter island is slightly less CpG dense than the other primates (18.7% in human, 19.1% in chimpanzee and 19.3% in rhesus macaque), so from this could be expected to be more, not less, methylated. The total sequence similarity is 98.3% between human and chimpanzee and 96.3% between human and rhesus macaque. We examined the comparative TRANSFAC TFBS motif analysis via TRAP for the *LTB4R*-associated s-DMRs. This revealed no significant difference in known MDR’s CTCF or SP1 motifs in this region. The only highly significant human-specific gain of binding affinity within this island was for Rfx1 (Regulatory factor X1, aka Enhancer factor C, V$ECF_Q6), with a significant human motif binding *P*-value of 0.042, and non-significant chimpanzee (*P* = 0.635) and rhesus macaque (*P* = 0.491) prediction (Additional file 7). As previously stated, Rfx2, another member of the RFX winged-helix transcription factor motifs, has been found to contribute to methylation variation in MDRs [27], although whether one motif would be expected to lead to such a considerable difference in methylation is not certain. Lienert *et al*. [27] identified an incremental additive effect of multiple MDR motifs required to modify methylation to this level, thus possibly implicating additional non-genetic or environmental factors in this locus. The RFX1 motif shows human-specific binding due to a central T present at chr14:24779978, which is the C of a CpG in all other primates. There is also no human common polymorphism at this site (dbSNP138). Furthermore, it is interesting to note that the modern human motif sequence is identical in both the archaic Denisovan hominin and the recent high coverage Altai Neanderthal sequences, thus dating this particular genetic change to at least prior to approximately 600,000 years ago [107,108].

#### Different LTB4R (BLT1) mRNA expression and signaling in human and rhesus macaque cells

**Figure 11:**
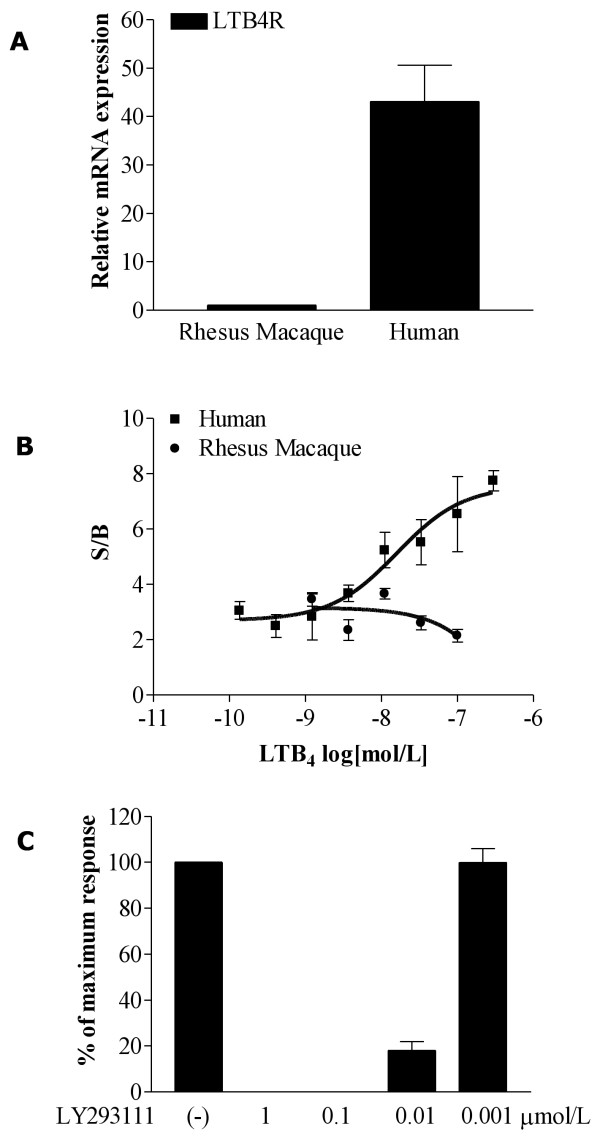
**Expression and signaling of LTB4R (BLT1) in PBMCs isolated from human and rhesus macaque peripheral blood. (A)** Real-time RT-PCR analysis of *LTB4R* mRNA in isolated human and rhesus macaque PBMCs normalized to the reference gene (18s); results from seven different human donors and from pooled rhesus macaque PBMCs run as four separate experiments; mean ± standard error of the mean. **(B)** Human and rhesus macaque PBMCs were stimulated with indicated concentrations of LTB_4_ and intracellular calcium mobilizations were recorded. Results are expressed as the ratio of stimulated over basal (S/B) peak calcium fluxes obtained from three different human donors and pooled rhesus macaque PBMCs run as four experiments analyzed simultaneously, mean ± standard error of the mean. **(C)** Human PBMCs were pre-incubated for 10 minutes with different concentrations of BLT1 inhibitor LY22398 and calcium mobilization was analyzed in response to LTB_4_ (300 nmol/L). Data are presented as mean ± standard error of the mean percentages of maximum response to LTB_4_ (N = 3).

To investigate the potential effects of the different epigenetic states of the *LTB4R* (*BLT1*) promoter, we performed comparative functional analysis between human and rhesus macaque PBMCs for differential mRNA expression and signaling. We evaluated the mRNA expression of *LTB4R* and the 18s gene as control using TaqMan primers and probes that are complementary to the gene sequence in both species. Similar levels of internal control 18s mRNA were observed in human and rhesus macaque PBMCs, while 40-fold higher levels of *LTB4R* mRNA were identified in human cells (Figure 11A).

LTB_4_ signaling through the LTB4R/BLT1 receptor induces calcium mobilization in cells known to express the receptor [58]. To determine whether the increased expression of *LTB4R* mRNA in human PBMCs resulted in functional expression of LTB4R/BLT1, human and rhesus macaque PBMCs were stimulated with different concentrations of LTB_4_ and intracellular calcium flux was measured (as described in Methods). Human and rhesus macaque PBMCs responded similarly to a non-specific calcium activator, the calcium ionophore A23187 (1 μmol/L). However, concentration-dependent responses to LTB_4_ were observed in human cells in contrast to rhesus macaque cells where no specific calcium mobilization was identified (Figure 11B).

The LTB4R/BLT_1_ selective antagonist LY223982 was used to confirm specificity of calcium responses and full inhibition of calcium mobilization in response to LTB_4_ was observed in human PBMCs (Figure 11C). These data support that the identified human-specific differential methylation pattern within the *LTB4R* locus may affect mRNA expression, signaling and function of the LTB4R/BLT1 receptor.

## Discussion

Human uniqueness has arisen due to the accumulation of genetic, environmental, behavioral and cultural changes [39,109]. Subtle variation within all these factors, separately and in complex combinations, also contributes to disease susceptibility. Additionally, the more extreme differences between species contribute to species-specific disease variation [110]. Higher prevalence of some common human diseases has been postulated, even whilst taking environmental differences into consideration, including Alzheimer’s disease, coronary artery disease and some cancers [111]. Humans have also been observed, comparatively, to have an excessively heightened inflammatory response, with genetic contributions identified, including *SIGLEC* genes [112,113].

By comparative tri-primate peripheral blood DNA methylation analysis we have identified strong DNA methylation variation between three primate species, human, chimpanzee and macaque, and located human-specific DMRs with potential regulatory consequences. Many regulatory loci are not constrained across mammalian evolution [52] and genetic loss of human-specific regulatory DNA has previously highlighted regulation change as a human evolutionary mechanism [17].

Both the hypomethylated and hypermethylated s-DMRs identified were skewed towards weak promoters, enhancers and transcribed regions. We also identified these DMRs to be more prevalent within moderately CpG dense CpGi shores than the islands themselves, consistent with tissue-, cancer- and reprogramming-specific changes [82,83]. Promoter hypomethylated regions that are common across diverse cell types are often found to possess a central region that remains unmethylated with an outer tidal region that shifts in a lineage-specific manner modulating associated gene expression [114]. DNA binding proteins localizing within these boundary regions, or shores, may help define these methylation states [18]. Thus, these species-specific modifications may be acting in similar fashion.

Pathogens have imposed a strong selective influence on the human genome [115]. Therefore, the unique evolutionary histories of these primates post-speciation would be expected to lead to unique immune systems. These strongly divergent differences have been recently exposed to be extreme within mammals, whereby the mouse was shown not to reproduce any of the patterns of gene expression induced by human inflammatory disease [116,117]. This poor model of human inflammation was found to perform no better than random. Human immunological variation is of particular interest due to the extreme rise of inflammatory diseases within the past few generations [118]. Combined genetic and new environmental factors may be driving this increase, with effects on immune development proposed, and epigenetic alterations are being explored to explain these immune system-related diseases [119].

We identified strong human-specific variation in *LTB4R*, important in the innate immune leukotriene and prostaglandin signaling pathway. Hypomethylated s-DMRs were identified covering both the promoter CpG island and shore region of this gene. In addition, inverse species-specific methylation was identified in the gene body of *LTB4R*. These significant DNA methylation differences identified in peripheral whole blood were replicated in neutrophil-only data, within both the promoter and the gene-body region. These data were also consistent with a H3K4me3 ChIP-seq study from primate lymphocyte cell-lines, with a strong active promoter signature present only in human. Comparative functional analysis also supported these large differences found across major blood cell types by showing human-specific increased expression and ligand response in peripheral blood mononuclear cells. These results are therefore consistent with the epigenetic ‘dimmer switch’ being dialed up in this human locus, in contrast to on/off human-specific regulatory deletions [17]. Furthermore, a recently published comparative array analysis in peripheral blood from Hernando-Herraez *et al*. [119], including further primate species (bonobo chimpanzee, gorilla and orangutan), supports the human-specific epigenetic state of the *LTB4R* locus. In this study three available complementary CpG dinucleotide positions located within the *LTB4R* promoter possessed human-specific hypomethylation.

The *LTB4*-*LTB4R* (BLT1) pathway, because of its significant immunological role, has been implicated in the pathophysiology of a number of diseases, including asthma (including airway hyper-responsiveness, severe attacks and asthma exacerbations) [97], atherosclerosis, Alzheimer’s disease [120], obesity-related inflammation [121], and inflammatory bowel disease [101]. LTB4R is involved in amplifying T-cell recruitment [122] and a heightened T-cell response in human compared with chimpanzee has previously been identified [123]. It is also of interest to note that up-regulation of *LTB4R* expression has been identified in seven out of nine experiments in human via the Expression Atlas [124] and furthermore that *LTB4R* expression on T cells is regulated by inflammation and may link innate and adaptive immune responses [122]. A number of further promoter associated s-DMRs would also be of interest for further follow-up, including *MAPK15* (mitogen-activated protein kinase 15), involved in autophagy [125], and the *MECP2* gene, which is mutated in Rett syndrome [126].

The effect of genetic mutation in particular TFBSs on the methylation state of CpGis has recently been refined by Lienert *et al*. [27]. Our identification of human divergent CTCF and RFX1 motif changes are consistent with these MDR data. These thus shift the genetic set point of methylation, within these promoters, between these primates. Additional environmental factors may then lead to further subtle variation.

Furthermore, from this comparative epigenomic work we propose the ‘s-DMR hypothesis’ whereby regions of significant epigenetic difference between humans and other primates that are highly conserved at the sequence level may include loci subject to, or indicative of, human-specific environmental influence, potentially in association with pathogenic conditions. Thus, focusing on these regions may enable the identification of endemic human environmental imprints on the epigenome.

## Conclusion

Comparison with our most closely related primate relatives enables insights into human-unique physiology. It is vital to identify and explain these differences, particularly as we readily extrapolate disease pathology from models in other species. Comparative epigenomics enables robust regulatory modifications to be pinpointed, and by integration with genetic data, a more complete functional picture can be elucidated. We have shown within blood tissue that the DNA methylome is not identically conserved between these primate species. These variations may be strongly driven by facilitative genetic means, but also potentially additional environmental factors. We have identified that the *LTB4R* gene is a significant human-specific epigenomic outlier, whilst containing minimal genetic differences. Thus, this finding reveals human-specific change in the innate immune system that may be a human-specific susceptibility factor, an inbuilt resultant common primate response to modern human conditions, or a combination of both, possibly contributing to the high level of these common diseases in the current environment.

## Abbreviations

bp: base pair; ChIP: chromatin immunoprecipitation; CpGi: CpG island; DMR: differentially methylated region; FDR: false discovery rate; GEO: Gene Expression Omnibus; L-DMP: leukocyte subtype-related differentially methylated CpG position; MC: Monte Carlo; MDR: methylation determining region; MeDIP: methylated DNA immunoprecipitation; MYA: million years ago; PBMC: peripheral blood mononuclear cell; PCR: polymerase chain reaction; s-DMR: species-specific DMR; SINE: short interspersed nucleotide element; SNP: single-nucleotide polymorphism; TFBS: transcription factor binding site; UCSC: University of California Santa Cruz.

## Competing interests

The authors declare that they have no competing interests.

## Authors' contributions

Conceived and designed the experiments: CGB, SB, GW. Performed the experiments: CGB, LMB, HRF, GW. Analyzed the data: CGB, GAW, GW. Contributed reagents/materials/analysis tools: CR, LW, GW, CGB, GAW, AF. Wrote the paper: CGB. All authors reviewed and approved the manuscript.

## Supplementary Material

Additional file 1: Table S1 Genomic Regions Enrichment of Annotations Tool (GREAT) s-DMR binomial analysis. Click here for file

Additional file 2: Figure S1 *Alu* distribution of human s-DMRs. Hypermethylated s-DMRs are depleted within both the most ancient *AluJ* and youngest *AluY* categories, but were enriched within the second oldest *AluS* set (χ^2^*P* < 2.2 × 10^-16^), which still possesses mobilization ability [77]. Hypomethylated s-DMRs also show this pattern, with an increase in *AluS* (χ^2^*P* < 2.2 × 10^-16^), but also an increase in the youngest and most active *AluY* (χ^2^*P* = 1.15 × 10^-4^). Click here for file

Additional file 3: Table S2 CpG island and CpG island shore s-DMRs. Click here for file

Additional file 4: Table S3 Transcription Factor Affinity Prediction (TRAP) motif results across species. Click here for file

Additional file 5: Table S4 Gene body methylation of 5′ promoter s-DMR genes. Click here for file

Additional file 6: Figure S2 BioGPS - human *LTB4R* expression profile. Click here for file

Additional file 7: Figure S3 Change in transcription factor motif binding prediction within s-DMRs between primates calculated via TRAP [55] for all 904 TRANSFAC motifs [56] within all individual hypomethylated s-DMR and hypermethylated s-DMR CpGi regions. The red lines indicate a magnitude increase or decrease in predicted motif binding (-log_10_*P*-value). In (A) the red circle in human out-lying positive motifs indicates the *LTB4R* - RFX (EFC_ Q6) motif. Click here for file
