# Supplementary figures and images for: Human-specific epigenetic variation in the immunological Leukotriene B4 Receptor (LTB4R/BLT1) implicated in common inflammatory diseases
Source: Genome Med. 2014 Mar 5;6(3):19. doi: 10.1186/gm536 (PMC4062055; doi:10.1186/gm536)

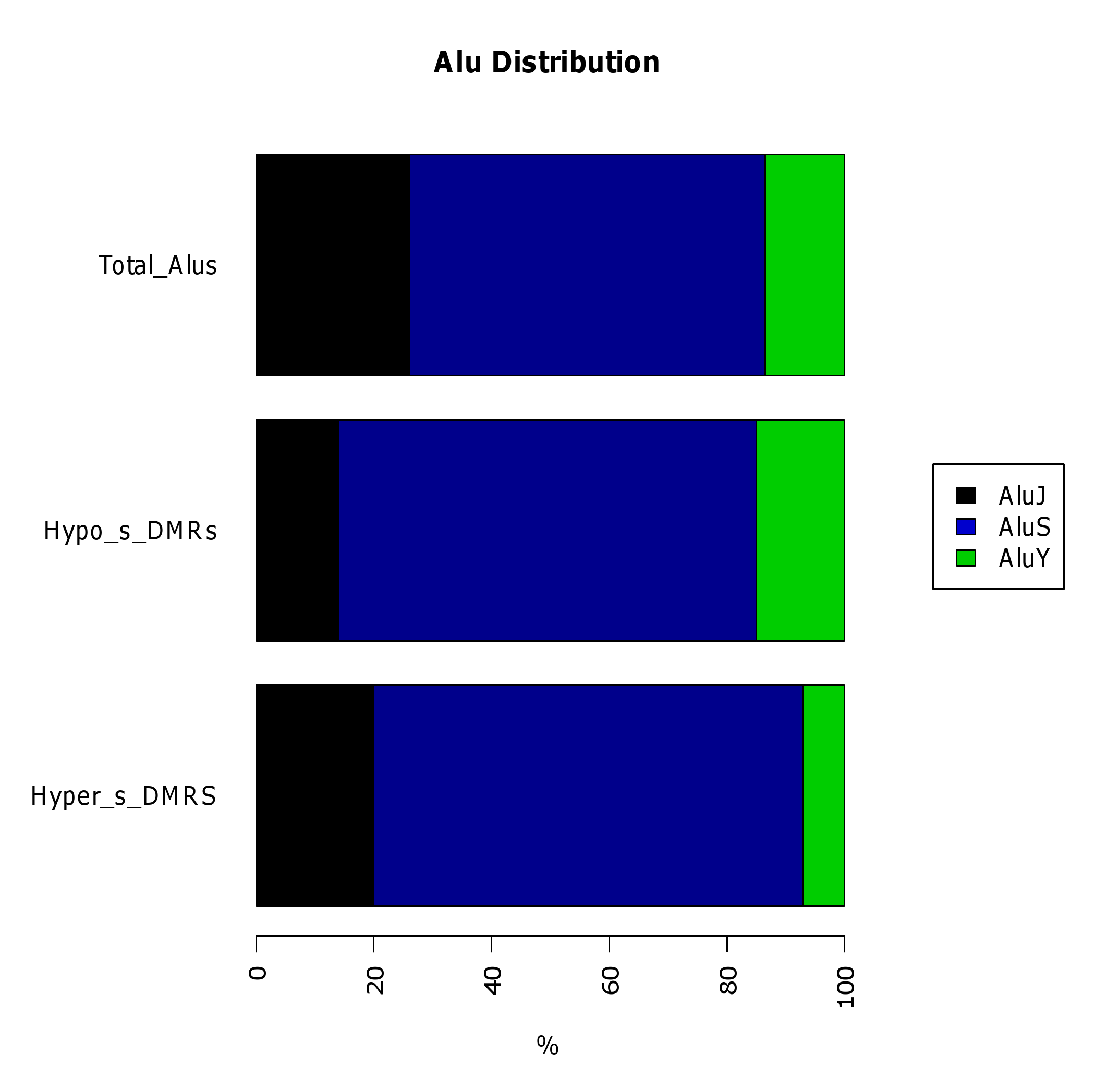

Supplement: Additional file 2: Figure S1 — Alu distribution of human s-DMRs. Hypermethylated s-DMRs are depleted within both the most ancient AluJ and youngest AluY categories, but were enriched within the second oldest AluS set (χ2P < 2.2 × 10^-16^), which still possesses mobilization ability [77]. Hypomethylated s-DMRs also show this pattern, with an increase in AluS (χ2P < 2.2 × 10^-16^), but also an increase in the youngest and most active AluY (χ2P = 1.15 × 10^-4^). [file gm536-S2.tiff]

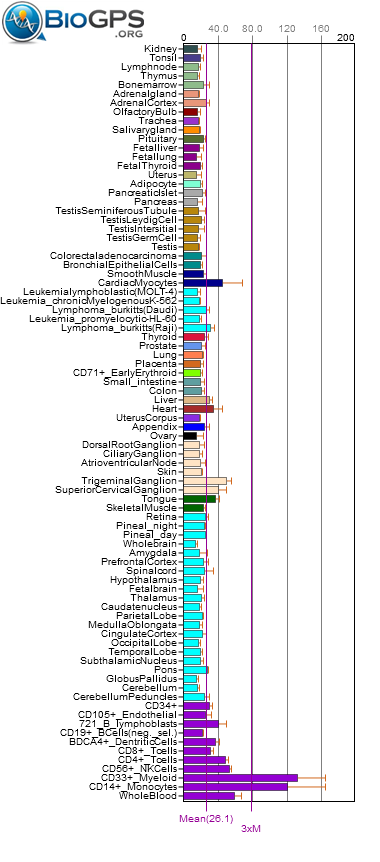

Supplement: Additional file 6: Figure S2 — BioGPS - human LTB4R expression profile. [file gm536-S6.tiff]

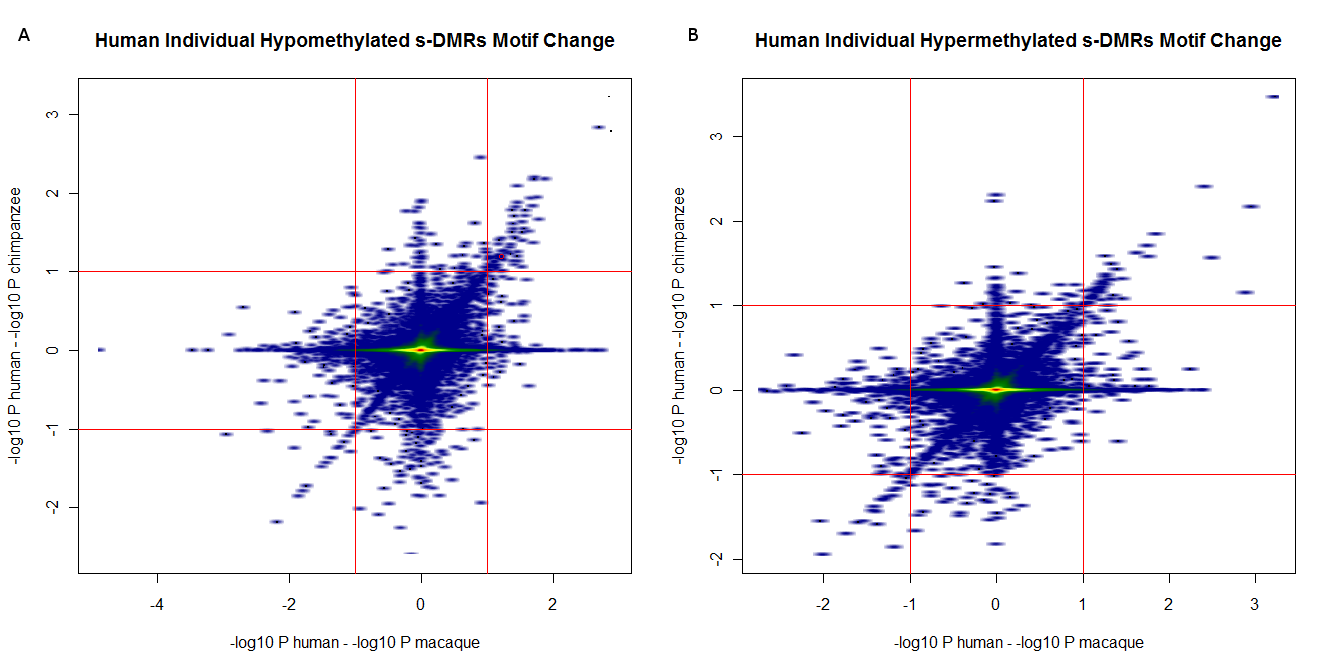

Supplement: Additional file 7: Figure S3 — Change in transcription factor motif binding prediction within s-DMRs between primates calculated via TRAP [55] for all 904 TRANSFAC motifs [56] within all individual hypomethylated s-DMR and hypermethylated s-DMR CpGi regions. The red lines indicate a magnitude increase or decrease in predicted motif binding (-log10P-value). In (A) the red circle in human out-lying positive motifs indicates the LTB4R - RFX (EFC_ Q6) motif. [file gm536-S7.tiff]
